# Fruchterman–Reingold Hexagon Empowered Node Deployment in Wireless Sensor Network Application

**DOI:** 10.3390/s22145179

**Published:** 2022-07-11

**Authors:** Jiahao Li, Yuhao Tao, Kai Yuan, Rongxin Tang, Zhiming Hu, Weichao Yan, Shiyun Liu

**Affiliations:** 1 School of Mathematics and Computer Science, Nanchang University, Nanchang 330031, China; 6002119021@email.ncu.edu.cn (J.L.); 401030919018@email.ncu.edu.cn (Y.T.); 2 Institute of Space Science and Technology, Nanchang University, Nanchang 330031, China; yanweichao@email.ncu.edu.cn; 3 School of Information and Engineering, Nanchang University, Nanchang 330031, China; 4 Jiangxi Provincial Key Laboratory of Interdisciplinary Science, Nanchang University, Nanchang 330031, China; 5 Department of Physics, Nanchang University, Nanchang 330031, China; huzhiming@caict.ac.cn; 6 China Academy of Information and Communications Technology (Jiangxi) Science and Technology Innovation Research Institute Co., Ltd., Nanchang 330031, China; 7 Usun Miscroelectronics, Nanchang 330072, China; mackliu@usunmicro.com

**Keywords:** wireless sensor network, Fruchterman–Reingold algorithm, node deployment, unmanned aerial vehicle

## Abstract

Internet of Things (IoT) and Big Data technologies are becoming increasingly significant parts of national defense and the military, as well as in the civilian usage. The proper deployment of large-scale wireless sensor network (WSN) provides the foundation for these advanced technologies. Based on the Fruchterman–Reingold graph layout, we propose the Fruchterman–Reingold Hexagon (FR-HEX) algorithm for the deployment of WSNs. By allocating edges of hexagonal topology to sensor nodes, the network forms hexagonal network topology. A comprehensive evaluation of 50 simulations is conducted, which utilizes three evaluation metrics: average moving distance, pair correlation diversion (PCD), and system coverage rate. The FR-HEX algorithm performs consistently, the WSN topologies are properly regulated, the PCD values are below 0.05, and the WSN system coverage rate reaches 94%. Simulations involving obstacles and failed nodes are carried out to explore the practical applicability of the FR-HEX algorithm. In general, the FR-HEX algorithm can take full advantage of sensors’ hardware capabilities in the deployment. It may be a viable option for some IoT and Big Data applications in the near future.

## 1. Introduction

**Figure 1:**
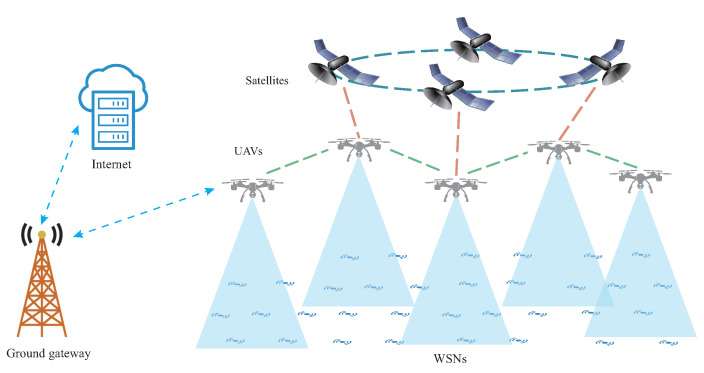
A typical application scenario for space–air–ground collaborative system consisting of satellites, unmanned aerial vehicles (UAVs) and wireless sensor networks (WSNs).

Internet of Things(IoT) and Big Data technologies are becoming increasingly significant parts of national defense and the military, as well as in the civilian usage [1,2,3,4]. Widely deployed wireless sensor networks (WSNs) have been collecting enormous data from industry, urban areas and the natural environment in last decades. With the increasingly advanced IoT and Big Data technologies, WSNs are becoming larger and more complex. In recent years, the utilization of collaborative drone-wireless sensor network (UAV-WSN) systems is becoming increasingly prevalent [5]. As shown in Figure 1, these systems integrate data from ground (WSN), air (UAV), space (satellite) and internet. They have been shown to be effective in large-scale surveillance with high mobility, high accessibility, and low emergency response time [6]. Well-deployed WSNs are the basis of efficient data collection in the region of interest and later tasks.

In common application scenarios, sensor nodes are often placed at random in the working space. Such a strategy will lead to low coverage and lower monitoring quality [7,8]. An appropriate node deployment strategy can improve WSNs’ coverage and maximize their energy efficiency [9,10]. In the field of WSN deployment, the particle swarm optimization (PSO) algorithm is frequently used and has proved to be a valid method. In [11], Zhang et al. proposed an artificial immune particle swarm optimization algorithm that synthesized the strength of PSO and artificial immune mechanism to solve the k-coverage problem with ideal convergence. Abba et al. formulated the mobile sensing problem like a non-linear optimization problem and proposed bacterial foraging optimization that allows mobile sinks to move in a self-organized and self-adaptive way to improve the network coverage [12]. In [13], the fruit fly optimization algorithm was introduced to eliminate overlapping nodes while saving the nodes’ energy. Qasim et al. used a modified ant colony optimization algorithm to achieve ideal coverage in a low computational cost way [14]. In [15], a distributed hybrid artificial fish swarm algorithm that takes water flow into consideration was proposed to improve the net work coverage efficacy. However, for large-scale WSN deployment, these algorithms are difficult to get rid of the local minima as the optimization problem becomes more complicated.

Another novel approach for deploying WSNs is the virtual force algorithm (VFA). VFA has a wide range of adaptability, especially in the scene of sparse node density in the monitoring area. In view of the general virtual force algorithm’s shortcomings, such as uneven distribution of nodes and more overlap of coverage area, Liu et al. [16] proposed a virtual molecular force algorithm to maximize the coverage of network. Qi et al. proposed a novel mobile-coverage scheme to cover the target region with a minimal number of mobile sensor nodes [17]. Inspired by the dust plasma crystallization phenomenon, Tang et al. proposed a 3D deployment algorithm and investigated the parametric effects of the Debye length for network coverage [18]. In [19], Liu et al. proposed a distributed node deployment algorithm based on virtual forces to improve network coverage, reduce node energy consumption and balance node residual energy. In [20], Deng et al. proposed an improving strategy, which incorporated external central forces to aid hexagonal topology development. When the number of nodes is small, the generic virtual force algorithm can uniformly cover the monitoring region. When the number of nodes in the monitoring area is large, however, the monitoring area’s peripheral nodes will push the monitoring area’s center nodes out of the way, resulting in an unequal distribution of nodes in the monitoring area.

Graphs are frequently utilized in computer science for social network analysis, computer networks, transportation networks, and many other applications. Layout algorithms are used to aid readers in comprehending the information of graphs [21]. To form an aesthetically pleasing graph, layout algorithms arrange the vertices of a graph so that all of the edges are about equal in length and there are a few crossing edges. The deployment of WSN resembles forming a graph with a minimum number of sensors to cover the maximum area. The Fruchterman–Reingold algorithm is one of the most well-known graph layout algorithms. It specifies the optimal distance between each vertex according to their edge connections. Taking the sensor as the graph’s vertex, the Fruchterman–Reingold algorithm can be used as a deployment algorithm for WSN. The large-scale WSN topology can be controlled through edge connections. In this way, the uneven distribution of nodes in center and periphery can be eliminated.

In this work, we modify the Fruchterman–Reinglod algorithm to be suitable for the deployment of WSN. To make the network form a regular hexagonal topology, we propose an edge allocation strategy, which takes energy consumption in to account. Thus, we develop the Fruchterman–Reingold hexagon (FR-HEX) algorithm. We introduce three evaluation metrics, such as average moving distance, pair-correlation diversion (PCD), and system coverage rate. We adopt these three evaluation metrics to comprehensively evaluate 50 simulations. We further explore the performance of the FR-HEX algorithm in such situations as obstacles occurred in the target area and nodes failed.

The rest of the manuscript is organized as follows: Section 2 proposes the Fruchterman–Reingold hexagon algorithm. Section 3 introduces three evaluation metrics. Section 4 and Section 5 present the simulation results, and Section 6 and Section 7 conclude the paper.

## 2. Implementation of Fruchterman–Reingold Hexagon Algorithm

The Fruchterman–Reingold algorithm is based on the theory of particle physics. It follows two simple principles: nodes connected with edges should be close to each other; other nodes should not be too close to each other [22]. The algorithm calculates the displacement of the nodes by the interactions of attractive and repulsive forces between the atoms. Following the law of motion similar to that of atoms or planets, the system eventually enters a state of dynamic equilibrium.

### 2.1. Modification of Fruchterman–Reingold Algorithm

In the Fruchterman–Reingold algorithm, the displacement between sensor nodes is calculated by these two functions.

(1) $$\begin{array}{c} f_{r}\left(d\right)=\frac{k^{2}}{d} \end{array}$$

(2) $$\begin{array}{c} f_{a}\left(d\right)=-\frac{d^{2}}{k} \end{array}$$

In Functions (1) and (2), $k=\sqrt{a^{2}/\left(1+N\right)}$ denotes the optimal distance of nodes in area *a* (*N* denotes the number of nodes), and *d* denotes the distance between nodes. Function $f_{r}$ works as repulsion, while function $f_{a}$ acts as attraction. When d=k, it is easy to deduce that $f_{r}\left(d\right)+f_{a}\left(d\right)=0$, which suggests these two functions will maintain the node’s distance around *k* in the deployment.

WSNs’ primary task is to collect data in the target region. The distance between nodes can be maintained by functions $f_{r}$ and $f_{a}$, but controlling where nodes cluster is challenging, and therefore, a function $f_{g}$ similar to global gravity can be applied to help nodes cluster toward the target area. For nodes in the repulsive dominant range (where *k*, *d* are of the same order of magnitude and d<k), according to Functions (1) and (2), it is easy to deduce that $f_{r}\left(d\right)$ and $f_{a}\left(d\right)$ have the same order of magnitude as *k*. To keep $f_{r}$ dominant in the range d<k, $f_{g}$ needs to be on the same order of magnitude as $f_{r}\left(d\right)$ and $f_{a}\left(d\right)$. $f_{g}$ needs to maintain the ability to bring nodes to the center area, and the value of $f_{g}$ needs to vary with the distance between the nodes and center. So $f_{g}$ is defined as the following.

(3) $$\begin{array}{c} f_{g}\left(d_{0}\right)=-d_{0} \end{array}$$

The variable $d_{0}$ in $f_{g}$ has a different meaning than the variable *d* in Functions (1) and (2). The variable $d_{0}$ in $f_{g}$ is the distance between the node and the center.

Initially, each node is assigned a random position, and the core step is an iterative process. The node updates its position by calculating the displacement generated by $f_{r}$, $f_{a}$, $f_{g}$. A large moving distance in a step may cause the node to miss the equilibrium position. So some restrictions are made on the maximum moving distance of each step. After experiments, the maximum moving distance of each step can be set as $d_{max}=\left(k/2\right)\times 10^{-1}$. During the deployment of large-scale WSN, the functions $f_{r}$ and $f_{a}$ applied to a node need to be accumulated multiple times. The displacement of a node in a step becomes difficult to obtain within $d_{max}$. To further control the moving distance, a scaling parameter $\mu =1\times 10^{-2}$ is set to maintain the moving distance two orders of magnitude smaller than *k*. Assuming the sensors are mobile sensors with mobile devices or can be transported to a specified location via UAV, the location of sensor nodes can be obtained by GPS. (x,y) in the two-dimensional coordinate system is used to denote the position of sensor node. The final moving distance of node *i* in one step can be calculated by the following equations.

(4) $$\begin{array}{c} \Delta x=\sum_{j\in N,j\neq i}\left[\frac{d_{{\left(i,j\right)}_{x}}}{d_{\left(i,j\right)}}\left(f_{r}\left(d_{\left(i,j\right)}\right)+f_{a}\left(d_{\left(i,j\right)}\right)\right)\right]+\frac{d_{{\left(i,0\right)}_{x}}}{d_{\left(i,0\right)}}f_{g}\left(d_{i,0}\right) \end{array}$$

(5) $$\begin{array}{c} \Delta y=\sum_{j\in N,j\neq i}\left[\frac{d_{{\left(i,j\right)}_{y}}}{d_{\left(i,j\right)}}\left(f_{r}\left(d_{\left(i,j\right)}\right)+f_{a}\left(d_{\left(i,j\right)}\right)\right)\right]+\frac{d_{{\left(i,0\right)}_{y}}}{d_{\left(i,0\right)}}f_{g}\left(d_{i,0}\right) \end{array}$$

(6) $$\begin{array}{c} \Delta x_{\mu }=\mu \times \Delta x \end{array}$$

(7) $$\begin{array}{c} \Delta y_{\mu }=\mu \times \Delta y \end{array}$$

(8) $$\begin{array}{c} \Delta d=\sqrt{\Delta x_{\mu }^{2}+\Delta y_{\mu }^{2}} \end{array}$$

(9) $$\begin{array}{c} x=x+\Delta x_{\mu }\times \frac{\Delta d_{max}}{\Delta d} \end{array}$$

(10) $$\begin{array}{c} y=y+\Delta y_{\mu }\times \frac{\Delta d_{max}}{\Delta d} \end{array}$$

In the above equations, $d\left(i,j\right)=\sqrt{{\left(x_{i}-x_{j}\right)}^{2}+{\left(y_{i}-y_{j}\right)}^{2}}$, $d{\left(i,j\right)}_{x}=x_{i}-x_{j}$ ($d{\left(i,j\right)}_{y}$ uses the same calculation method), and *N* denotes the number of all nodes. The repulsive forces between all nodes are calculated, and for each node, the attraction forces generated by the nodes associated with it are calculated. The algorithm can be ended when all nodes’ positions do not change or when the iterations exceed a preset threshold.

### 2.2. Strategy of Edges Allocation

In two dimensions, a regular hexagon can fill the entire plane without any gaps or overlaps, and a regular hexagonal topology has the maximum coverage. To deploy WSN, we can refer to regular hexagon topology.

**Figure 2:**
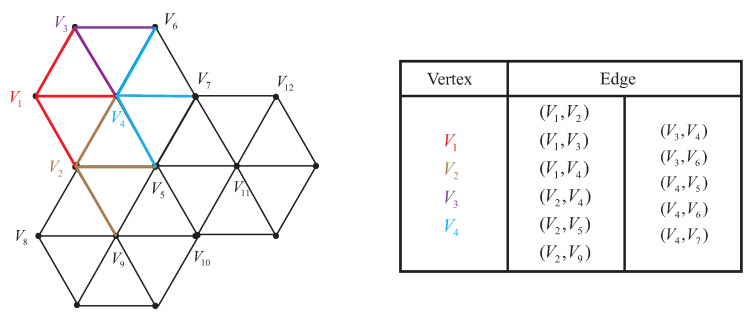
Composition of the edges of the vertices of a hexagonal topology, where edges of the same color are owned by a single vertex ($V_{1},V_{2},V_{3},V_{4}$ as examples).

**Figure 3:**
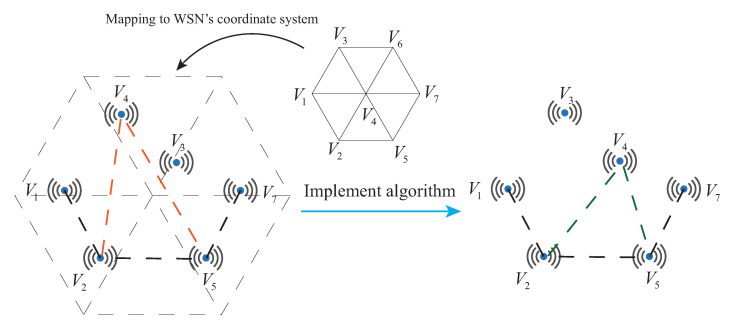
Edges formed through the nodes target vertices, before and after applying Algorithm 1.

Edges of vertices of regular hexagonal topology are defined like Figure 2. Vertices are connected by undirected edges, so that the attraction and repulsion between nodes will not be calculated twice in one step. One sensor node can correspond to one target vertex. For example, if the target vertex of node $S_{i}$ is $V_{i}$, edges in the Edge table held by $V_{i}$ can be allocated to node $S_{i}$. Then the Fruchterman–Reingold algorithm enables nodes to form a hexagonal network. However, if target vertices are randomly assigned to a node, there may be situations where nodes need to travel very large distances to get together. To avoid this situation, vertices of a hexagonal topology can be mapped to the coordinate system of the nodes. As shown in Figure 3, according to Algorithm 1, each node will find its optimal target vertex. Applying Algorithm 1 can minimize the intersection of edges between nodes. In this way, the overall travel distance of nodes in WSN is reduced when applying the Fruchterman–Reingold algorithm. We define the Fruchterman–Reingold hexagon (FR-HEX) algorithm as a method to determine the edge connection between the nodes and to deploy WSN close to a hexagonal structure using the Fruchterman–Reingold algorithm.
**Algorithm 1:** Find node’s best target vertex
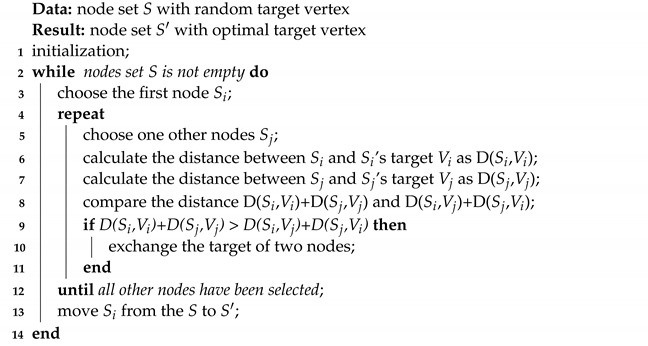


## 3. Evaluation Metrics for FR-HEX Algorithm

### 3.1. Average Moving Distance

In practice, sensors are usually powered by batteries, and the main energy consumption of nodes during deployment is movement. Because there is finite energy, the amount of energy utilized during deployment will have an impact on the node’s performance in later tasks. Thus the moving distance is utilized to determine the node’s energy consumption.

(11) $$\begin{array}{c} \bar{M_{t}}=\frac{1}{N}\sum_{i=1}^{N}d_{\left(S_{i(t)},S_{i(t-1)}\right)} \end{array}$$

In Equation (11), ${\bar{M}}_{t}$ denotes the average moving distance of the network at step *t*, $S_{i}\left(t\right)$ denotes the location of node *i* at step *t*, and $S_{i}\left(t-1\right)$ denotes the location of node *i* at step t−1.

### 3.2. Pair Correlation Diversion

The quality of network topology will affect the coverage of network and information transmission efficiency. However, it is difficult to evaluate the quality of topology by numerical methods. According to [23,24], the pair correlation Function (5) defines the probability of finding a particle at a distance *r* from the first particle at the origin. It can be used to depict the distribution of nodes in a topology. The resemblance of two topologies can be determining by comparing the pair correlation functions, and the smaller the difference between the pair correlation functions, the more similar the two topologies are. In two dimensions, hexagonal topology is optimal, so a regular hexagonal network topology is chosen to compare with the deployed network structure.

(12) $$\begin{array}{c} g\left(r\right)=\frac{N(r,\Delta )\times S}{2\pi r\Delta \times N} \end{array}$$

(13) $$\begin{array}{c} \delta \left(\Omega ,\Omega_{H}\right)=\frac{\int_{0}^{r_{T}}\left|\right|g_{\Omega }\left(r\right)-g_{\Omega_{H}}\left(r\right){\left|\right|}^{2}dr}{\int_{0}^{r_{T}}\left|\right|g_{\Omega_{H}}\left(r\right){\left|\right|}^{2}dr} \end{array}$$

Here, N(r,Δ) denotes the number of particles within the range (r+Δ/2,r−Δ/2) at *r* from the center of the system, *S* is the total area of the network, *N* is the total number of nodes in the network, and Δ is the average distance of the nodes. $\Omega_{H}$ in the equation that denotes the regular hexagonal topology, and Ω denotes the topology of network produced by our deployment algorithm. $r_{T}$ in the equation denotes the range of the regular hexagonal topology. $\delta (\Omega ,\Omega_{H})$ denotes the difference between the radial distribution function of the topology obtained by our deployment algorithm and the regular hexagon structure, that is pair correlation diversion (PCD).

### 3.3. System Coverage Rate

The most critical criterion in the deployment of WSN is coverage. The higher the coverage, the more comprehensive the information that the nodes will collect. The perception model used in this paper is the Boolean perception model. The probability that node *s* will perceive particle *p* is shown in the Equation (12). When a particle enters the node’s sensing radius ($R_{S}$), it is considered able to be monitored.

(14) $$\begin{array}{c} f\left(d_{\left(s,p\right)}\right)=\left\{\begin{array}{c} 1,d_{\left(s,p\right)}\leq R_{S}\\ 0,\;\mathrm{otherwise} \end{array}\right. \end{array}$$

**Figure 4:**
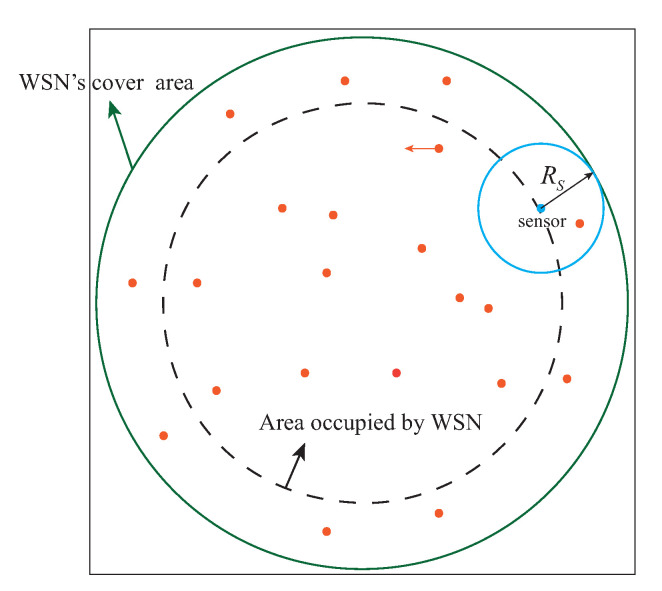
Coverage model of the entire WSN ($R_{S}$ denotes the sensing radius of the node and each particle denotes the target point to be detected).

As shown in Figure 4, there are *m* particles in the area covered by WSN (*m* is large enough to fill the entire area), and each particle is assumed to be a target point. In the area covered by WSN, sensor set *S* consists of *N* nodes, and the *i*th node in the set as $S_{i}$. Set the probability that the *j*th particle $p_{j}$ will be perceived in *S* to be $P(S,p_{j})$, and system coverage rate *C* can be expressed as the following equation.

(15) $$\begin{array}{c} P\left(S,p_{j}\right)=1-\prod_{i=1}^{N}(1-f\left(d_{\left(S_{i},p_{j}\right)}\right) \end{array}$$

(16) $$\begin{array}{c} C=\frac{\sum_{j=1}^{m}P\left(S,p_{j}\right)}{m} \end{array}$$

## 4. Simulation Analysis of FR-HEX Algorithm

**Figure 5:**
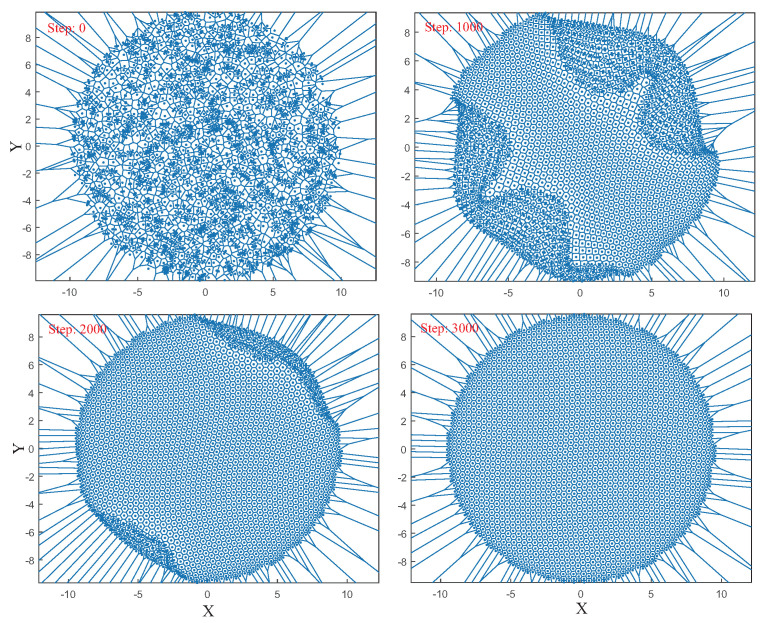
Voronoi diagram of the network topology with iterations of the FR–HEX algorithm.

We mainly focus on the performance of the algorithm in two-dimensional large-scale node deployment. We used Java code to implement the FR-HEX algorithm’s main iteration process (Equations (4)–(10)) and Algorithm 1. The version of the Java Development Kit is openjdk-16, and the cpu we used is Intel(R) Core(TM) i5-9500 CPU. In the following simulations, we randomly generate *N* = 2000 coordinates (denote the sensor nodes’ positions) in a circular area of radius r=a/2=5, which means for each node $x^{2}+y^{2}<r^{2}$. We can also obtain $k=\sqrt[{}]{a^{2}/\left(1+N\right)}=0.224$. We input these random node coordinates into the Java program to determine the edges between the nodes by Algorithm 1, after which we input the coordinates of the nodes and number of iteration into the iterative program (Java code implemented Equations (4)–(10)). Then in each iteration, the $f_{r},f_{a}$, and $f_{g}$ of each node are computed to obtain its coordinates at the next iteration. In this way, the FR-HEX algorithm is applied to adjust the sensor nodes’ coordinates. The coordinates processed by code iteration are saved per 10 steps for further numerical analysis. Figure 5 shows how the network topology changes with the iterations of the FR-HEX algorithm. The Voronoi diagram is used here to visualize the topology of the network, where one point represents one node. We can observe that when using the FR-HEX algorithm to deploy the large-scale node, the position of nodes in the central part of the network is adjusted evenly first, and then the distribution of the peripheral nodes gradually becomes uniform. At roughly the 3000th step, the network’s topology becomes rather neat, with a structure similar to that of a regular hexagon. There is no noticeable uneven distribution of central and peripheral nodes.

**Figure 6:**
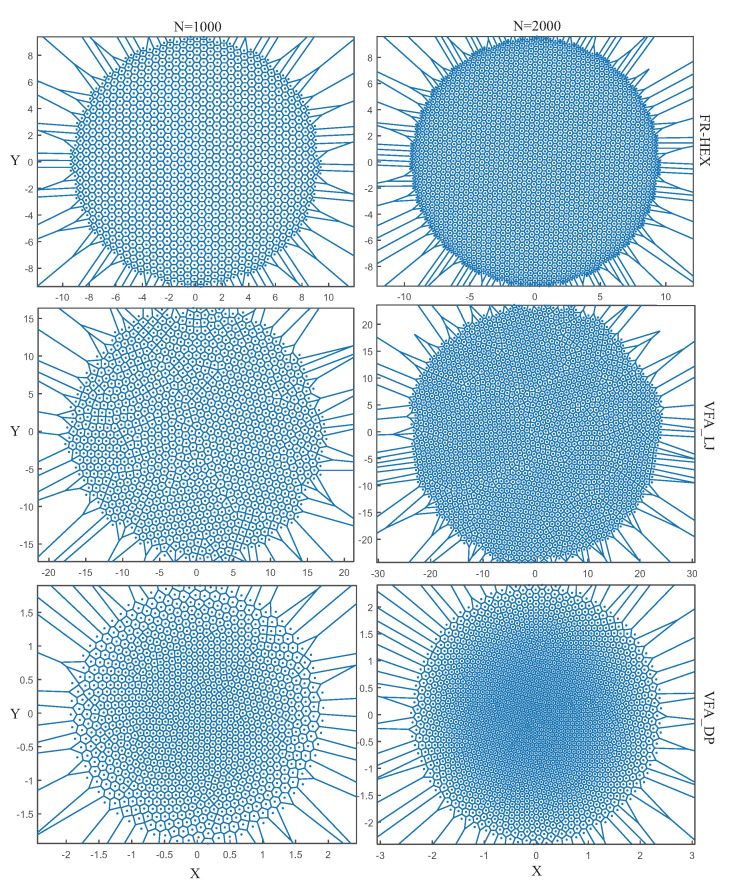
Voronoi diagrams of the network topology deployed by FR–HEX, VFA_LJ and VFA_DP at 3000th step in different scales (N=1000 and N=2000).

As shown in Figure 6, compared with the methods designed for large-scale WSNs, such as the virtual force algorithm inspired by Lennard Jones potential (VFA_LJ) and the virtual force algorithm inspired by Dusty Plasma crystallization (VFA_DP) [25], the topology produced by the FR-HEX algorithm is obviously more regular in different scale (N=1000 and N=2000). VFA_LJ always has some regions with uneven nodes. VFA_DP will result in the uneven distribution of nodes in central and peripheral parts, that is, nodes distributed tightly in the central part and loosely in the peripheral part. From Table 1, we can see at the 3000th step that FR-HEX has much smaller PCD values of 0.045 and 0.023. It numerically shows that the network deployed by the FR-HEX algorithm has a topology more similar to that of a regular hexagon. We can infer that the uneven network topology due to VFA_LJ and VFA_DP may be worsened when the deployment scale becomes larger as PCD goes up when *N* changes from 1000 to 2000. This will also cause some limitations on their application scenarios. However, the FR-HEX algorithm is almost unaffected by the scale change, with only a slight change in the PCD value.

To further explore the performance of the FR-HEX algorithm in deploying large-scale nodes, we performed 50 simulations. In each simulation, we still randomly generated 2000 coordinates in a circular area of radius r=5. For the 50 simulation experiments (iterations of 6000 steps), the average run time was 113.357 s with a standard deviation of 2.081 s. We employed an average moving distance ($\bar{M}$), pair correlation diversion (PCD) and system coverage rate (*C*) to comprehensively analyze the results and evaluate the FR-HEX algorithm.

For large-scale WSNs deployment, the complexity and execution time of the algorithm may cause some problems in real-world applications. Our FR-HEX algorithm is computationally efficient. For a node, it only needs to calculate the repulsive force $f_{r}$ of other nodes, and the attraction force $f_{a}$ of the node associated with it, and finally add the gravitational function $f_{g}$. We completed 50 simulations for different scale of nodes, respectively. As we can see in Table 2, we can observe a similar exponential increase in the execution time as the nodes go from 500 to 2000. However, for the case of 2000 nodes, which is relatively a large-scale WSN, the average execution time for 6000 iterations is only 113.357 s with a standard deviation of 2.081 s. Even if the number of nodes rises to 3000, the average execution time per step is only 0.066 s, which is acceptable. This shows that, in practice, the FR-HEX algorithm is able to deploy wireless sensor networks dynamically in a stable and efficient manner.

**Figure 7:**
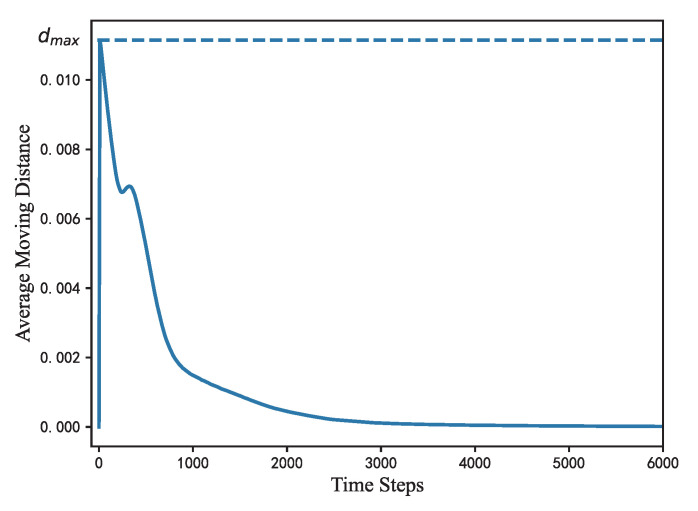
Mean value of the average moving distance of 50 groups of cases with FR-HEX algorithm iterations, $d_{max}$ denotes the maximum move distance restricted in one step.

Figure 7 shows the average value of the $\bar{M}$ per 10 steps of 50 cases in total 6000 steps. Since the nodes are randomly distributed in range r=5 at the start of the deployment process, the position of edge-related nodes needs to be adjusted through $f_{r}$ and $f_{a}$. Gravitational function $f_{g}$ also drives the nodes to converge toward the center. As a result, the movement of nodes is intense during the first 200 steps with $\bar{M}$ increased to 0.01. However, $\bar{M}$ is limited within $d_{max}=\left(k/2\right)\times 10^{-2}\times I_{s}=0.011$ ($I_{s}$ denotes the data interval, for nodes saved per 10 steps $I_{s}=10$). When the edge-related nodes have reached a relatively stable location, with attraction $f_{a}$ and repulsion $f_{r}$ and gravitation $f_{g}$ in a roughly balanced state, the nodes’ movement slows down gradually with $\bar{M}$ drop to 0.002 at the 1000th step. After reaching a rather steady distance under the simultaneous action of $f_{r}$, $f_{a}$ and $f_{g}$ at roughly 3000th step, the nodes will not move significantly with $\bar{M}$ close to 0. From the trend of the curves, it can be inferred that all cases have a reasonably constant change in average moving distance with no dramatic fluctuations.

**Figure 8:**
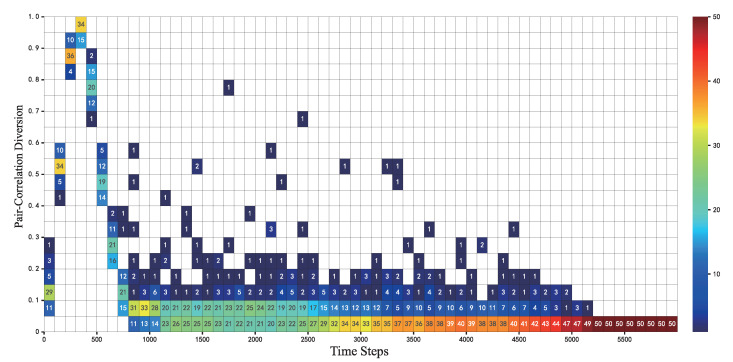
The PCD value of 50 cases changes with the iteration of FR-HEX algorithm, and the color bar indicates the number of cases in the interval of a certain PCD value.

In Figure 8 a heat map is used to show the distribution of PCD values over time for the 50 cases. The closer the color to dark red means the more cases with PCD values in this interval. A trend similar to that in Figure 7 can be observed. In the first 200 steps, when nodes adjust their distances based on edge connections, the PCD values change dramatically. At the 300th step, the PCD values of all cases are larger than 0.9. Then at the 600th step, 50 cases’ PCD values rapidly drop to 0.4. At 1000 to 2500 steps, it can be noticed that about 50% of the cases are able to reach a PCD value below 0.05. It can be inferred from Figure 7 that the nodes are still moving at this stage and the network topology is still adjusting. Very few cases have some fluctuations in PCD values due to the movement of a few nodes when adjusting the network structure, but such fluctuations can be adjusted quickly. At step 4500, all cases have PCD values within 0.2. At this point, the $\bar{M}$ is also reduced close to 0. Thereafter, there is basically no fluctuation in the position of the nodes. The PCD values of 50 cases are less than 0.05 at step 5200. The algorithm is starting to converge at this time, and the topology has stabilized.

The PCD value represents the resemblance between WSNs’ topology and regular hexagon topology on the one hand, and the algorithm’s convergence on the other. In practical applications, the algorithm can be judged to converge according to the change of PCD value. The operation of the algorithm can be terminated after the algorithm converges to reduce the computational cost.

If the average distance between well-deployed nodes is determined, the appropriate sensing radius for the sensor can be set based on the distance between nodes. This allows us to make the fullest use of the sensor’s hardware. All functions in FR-HEX, including $f_{r}$, $f_{a}$ and $f_{g}$, are designed relevant to *k*. In the original Fruchterman–Reingold algorithm, $f_{r}$ and $f_{a}$ maintain the distance between nodes at *k*. However, when function $f_{g}$ is added and the nodes are allocated with hexagonal edges, the force between nodes gets complicated, and the distance between nodes does not remain at *k*.

To determine the relationship between the average distance $d_{a}$ and *k*, we construct networks with 100, 500, 1000, 1500, and 2000 nodes in the range of a=10 and a=100 in our simulation experiments, respectively. Table 3 shows the relationship between $d_{a}$ and *k* value.

Changing the deployment range *a* does not affect the ratio of $d_{a}$ to *k*; increasing nodes will lead to an increase in $d_{a}$ between nodes. In the FR-HEX algorithm, functions $f_{r}$ and $f_{a}$ play a leading role in controlling the distance between nodes. Changing the value of deployment range *a* is equivalent to scaling $f_{r}$ and $f_{a}$ by the same multiple at the same time, but will not significantly affect the ratio of $d_{a}$ to *k*. When the number of nodes is increased, the number of times each node must accumulate repulsion $f_{r}$ increases, but there is no obvious rise in the accumulation number of $f_{a}$. This causes the repulsion force increases. At this time, the ratio of $d_{a}$ to *k* becomes larger.

**Figure 9:**
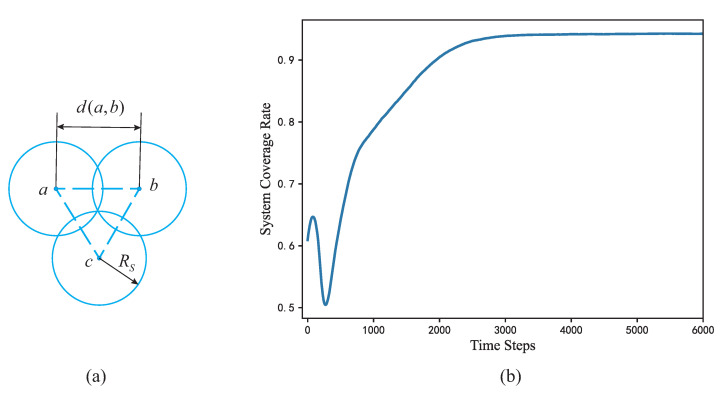
(**a**) Ideal coverage model for nodes (d(a,b) denotes the distance between node *a* and *b*, and $R_{S}$ denotes the sensing radius of the node), (**b**) mean value of system coverage rate for 50 cases with FR-HEX algorithm iterations.

As shown in the Figure 9a, to achieve the densest coverage between nodes, the relationship between the distance d(a,b) and the sensing radius of the sensor is $d\left(a,b\right)\leq \sqrt{3}R_{S}$ [25]. According to the statistics in the table, for large-scale deployment, the $d_{a}$ is about 1.7k. That is, $d_{a}=d\left(a,b\right)=1.7k$, and the $d_{a}$ is close to $\sqrt{3}k$. To achieve dense coverage, we can set $R_{S}=k$.

**Figure 10:**
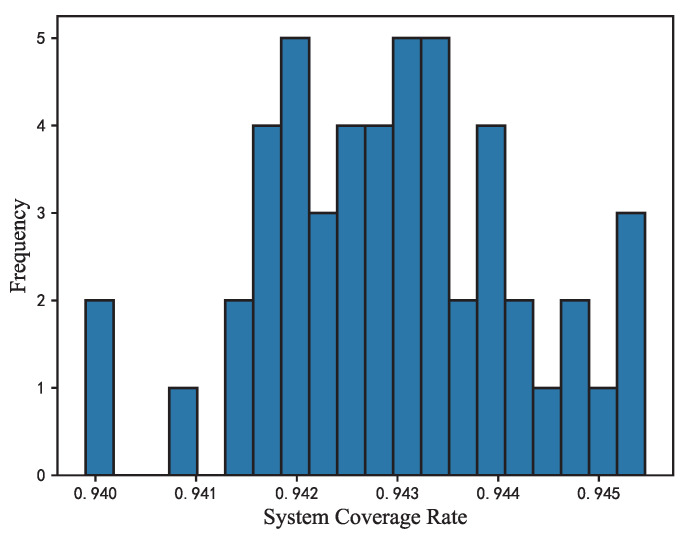
Frequency distribution of network’s system coverage rate for 50 deployments in convergence state.

According to the conclusion drawn above, we set $R_{S}=k$. Similar to the method used in Figure 7, Figure 9b shows the mean value of the *C* of 50 cases per 10 steps. The nodes move violently in the first 1000 steps. The *C* values have a obvious drop from 0.6 to 0.5 in the first 200 steps. The distance between the nodes become inconsistent as a consequence of location modification according to the edge connection. Inconsistent distance between nodes leads to reduced coverage. After the distance between nodes is adjusted at about the 2000th step, the network reaches a high coverage rate with C>90%. The *C* values of 50 cases are steady at around 4000 steps and begin to converge at the 5000th step with *C* values reaching about 94%, keeping the same trend as the preceding $\bar{M}$ (Figure 7) and PCD (Figure 8). Figure 10 shows the system coverage rate of 50 cases at the 6000th step. The  *C* values of 50 cases have reached about 94%, which is a relatively high coverage rate. When the algorithm converges, the system coverage rate of 50 simulations does not vary much such that all *C* values are within 0.940–0.945, demonstrating the stability of the FR-HEX algorithm to some degree.

The FR-HEX algorithm can effectively cover the target region by generating a hexagonal topology. The nodes connected by edges are relatively close together, which is ideal for covering the whole system, achieving high coverage of C>94%, and making efficient use of hardware resources. In practical application, the convergence of the algorithm can be judged according to the change of PCD value. We can apply the algorithm flexibly with the trend of system coverage rate. For cases where the monitoring accuracy requirement is relatively low, we can choose to terminate the algorithm at an early stage, such as 1000 steps, so that we can also achieve a *C* value of about 75%. However, for higher accuracy requirements, we can terminate the algorithm at around 5000 steps so that it ensures C>94%.

## 5. Simulation of Deployment with Obstacles and Failed Nodes

During the practical deployment process, some unexpected situations may occur. For example, before deployment, there are obstacles in the target area that may hinder the movement of nodes. Another situation is that after deploying nodes in the network may fail due to energy exhaustion or other reasons. Failed nodes may bring about blind areas for WSN monitoring and interrupt information transmission. We conduct simulations for both cases to explore the performance of the FR-HEX algorithm in these two situations.

### 5.1. Deploy Nodes in Areas with Obstacles

**Figure 11:**
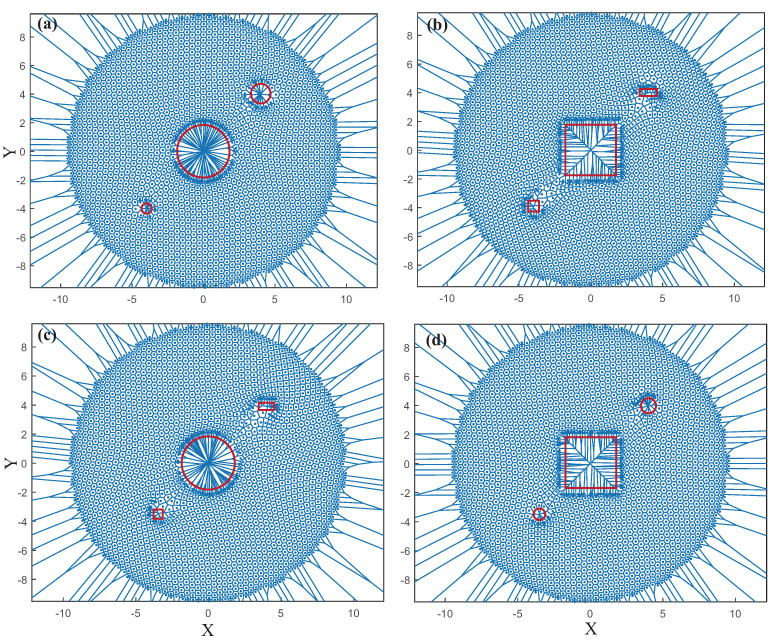
Network topology of WSN deployed by FR–HEX algorithm when there are obstacles in the target area. (**a**–**d**) The four possible scenarios of obstacles in the target area).

Obstacles may appear in the actual deployment, which will disrupt the network architecture and make data transmission challenging. Simulations are performed to explore the performance of FR-HEX when deploying WSNs with obstacles. As shown in Figure 5, under the action of function $f_{g}$, WSN is finally clustered in a circular area, which is highly symmetrical. Since all nodes have a tendency to move toward the center, obstacles in the center will have a greater impact on the overall topology, while obstacles at the edges have less impact on the network topology. So locations of the obstacles appeared in WSNs divide into two categories: central and peripheral. Obstacle types divide into rectangular and circular. The FR-HEX algorithm was put to the test in four different scenarios. As shown in Figure 11a–d, there are circular obstacles in the center and peripheral; rectangular obstacles in the center and peripheral; circular obstacles in the center and rectangular obstacles in the peripheral; and rectangular obstacles in the center and circular obstacles in the peripheral.

We can observe that when there is an obstacle in the center, whether the obstacle is circular or rectangular, the nodes can be evenly distributed around the obstacle. However, when there are obstacles at the peripheral area, the node distribution will be slightly uneven from left to right. From the comparison of (a) and (c), and (b) and (d), we can observe that the distribution of nodes in the region between the periphery and the center becomes more sparse when a rectangular obstacle appears at the periphery. For obstacle in the center, due to the function $f_{g}$ that moves the node to the center, the nodes can be evenly distributed around it, but the force on the nodes around the peripheral obstacle is very complex, resulting in the uneven distribution of nodes around the obstacle. Rectangular obstacles are relatively more irregular, and the rectangular obstacles appearing at the periphery have a greater impact on the network topology than the circular ones. We may infer that rectangular obstacle has a greater impact than circular obstacle on algorithms such as FR-HEX that form circular topologies. However, for the FR-HEX algorithm, the overall local obstacles do not significantly affect the whole network, and it still maintains a relatively stable state.

### 5.2. Reconstruction of Network with Failed Nodes

Another situation that might occur during the deployment is node failure. Without intervention, the WSN monitoring quality may decline with blind areas brought about by some failed nodes. If the information transmission was interrupted due to the failed nodes, inconsistent position information will cause chaotic movement while applying the FR-HEX algorithm. Thus the regular network topology may be destroyed. It is expected that even if some nodes in the network fail, the network can still provide adequate coverage of the target region. In simulations, we randomly take 500 nodes as failed nodes and assumed that we can obtain their edge relations and numbers in the whole node set. We tested in the simulation experiments that changing the number of failed nodes does not affect the performance of the FR-HEX algorithm in reconstructing the network. Five hundred failed nodes in the network is a relatively extreme case for a WSN consisting of two thousand nodes, and more failed nodes appearing may be impractical. The effect of FR-HEX reconstructing the network can also be observed more clearly by choosing a relatively large number of failed nodes. For the FR-HEX algorithm, two strategies can be adopted to restructure the network when fail nodes appear. The first strategy is maintaining the *k* value constant (that is, the distance between nodes remains constant) and shifting the node to the center that ensures that the core region is well covered. The second strategy is recalculating $k=\sqrt{a^{2}/\left(N+1\right)}$ based on the number *N* of existing nodes, such that when there are failed nodes in the network, the distance between existing nodes increases, allowing us to cover the origin target region.

When there are failed nodes, for the network topology to stay regular, it will inevitably lead to edge reorganization. When reorganizing the edge, the Algorithm 2 can be applied. Then, implementing the FR-HEX algorithm will further adjust the position between nodes. In Algorithm 2, $N_{loss}$ represents the number of failed nodes. $S_{c}$ represents the node in the central part, and $S_{p}$ represents the node in the peripheral part.
**Algorithm 2:** Restructuring the network**Data**: Origin nodes set $S_{o}$**Result**: Nodes set with reorganized edge1Sort the nodes in $S_{o}$ by their distance from the center (in ascending order)2$S_{c}$ = the first $N_{loss}$ nodes in $S_{o}$ 3$S_{p}$ = all nodes in $S_{o}$ except $S_{c}$4$S_{c}^{\prime }$ = failed nodes in $S_{c}$5$S_{p}^{\prime }$ = remained nodes in $S_{p}$6exchange edges between nodes in $S_{c}^{\prime }$ and $S_{p}^{\prime }$7remove all failed nodes in $S_{o}$

**Figure 12:**
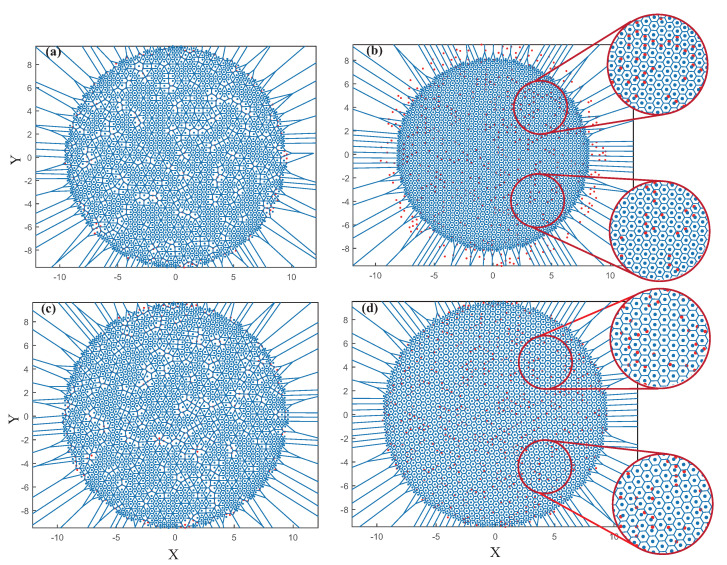
Topology reconstructed by FR–HEX algorithm when there are 500 failed nodes (red dots indicate failed nodes, (**a**–**b**) and (**c**–**d**) are two reconstruction strategies, respectively).

Figure 12 shows the network topology with 500 randomly failed nodes, where the red point represents the failed node. Figure 12b,d are the results of patching the vacancy in the network using the first and second strategy, respectively. From (a) to (b), we can observe that using the first method, the FR-HEX algorithm will force the remain node to replace the failed node. Peripheral nodes will gather to the center to guarantee that the target area’s center is covered. The network does not need to be adjusted greatly. The local topology is still a perfect hexagonal topology when other nodes make up for the failed nodes, as can be seen in the local magnified figure. From (c) to (d), we can observe that, following the execution of the algorithm, the distance between nodes will be readjusted according to the recalculated *k* value. As the number of nodes *N* becomes smaller, the value of *k* will correspondingly become larger. *k* is related to the distance between nodes in the network. When the value of *k* changes, this means that the relative positions of all nodes in the network have to change. In Figure 12d, we can see that eventually the remaining nodes can still cover the target area evenly due to the greater distance between them, but to achieve the original coverage, the sensing range of the sensor nodes needs to be adjusted.

The final network topology of fixing failed nodes using the two methods is ideal, including regular hexagonal topology. In practice, we may utilize the second way to cover the original target region if the node’s hardware is powerful enough to modify the sensing radius. If its hardware resources are limited, we may use the first method to ensure that the center area is covered.

## 6. Discussion

Based on the Fruchterman–Reingold algorithm that uses $f_{r}$ and $f_{a}$ to adjust the graph’s vertices, we apply the Fruchterman–Reingold algorithm to deploy WSNs. Additionally, $f_{g}$ is added to make it more suitable for large-scale WSN deployment. To form the hexagonal topology, we propose an edges allocation strategy. Combining above modification and method, the FR-HEX algorithm is developed. Three kinds of evaluation metrics, such as average moving distance, pair correlation diversion (PCD), and system coverage rate, are introduced to numerically assess the algorithm. In the simulation, the FR-HEX algorithm runs stably and has excellent convergence. The network topology deployed by the algorithm is very close to the regular hexagon topology with PCD value below 0.05. The network’s system coverage rate reaches about 94%. In practice, these evaluation metrics can also be used as a reference for the convergence of the FR-HEX algorithm, allowing for flexible application of the algorithm. The FR-HEX algorithm performs well in situations such as obstacles in the target area and failed nodes. When obstacles appeared the FR-HEX algorithm nevertheless enables nodes to retain an ideal overall network topology. When nodes failed, we may choose the strategy for restructuring the network according to the hardware. If the node can change the sensing radius, the remaining nodes can be used to cover the original target area. If the node’s hardware is poor, nodes may shift to the center to cover the center area.

## 7. Conclusions

IoT is significant source for Big Data, and WSNs are the basis for IoT to collect and generate data. The future trend of IoT is more complex and larger in scale. For some special large-scale application scenarios in deep space or deserts that are unreachable for humans, AI anomaly detection in some industries, and online intelligent environment detection, WSNs need to have some self-organization capability. A good WSN structure ensures comprehensive collection of data in the target area as well as efficient transmission. The FR-HEX algorithm provides a solution for the dynamic deployment of WSNs in such applications. It will provide a more stable WSN structure for larger scale IoT, thus providing a reliable data source for Big Data application. Our algorithm can be applied in practice in two ways: one is to calculate the final deployment location in advance and deploy the sensor nodes to the corresponding locations using UAVs or other transportation devices. The second one is to optimize the existing network and dynamically adjust the location of the nodes in the network. The flexible deployment approach allows the FR-HEX algorithm to play an important role in future IoT and Big Data applications. This work focuses on the location deployment of sensor nodes in an ideal situation and does not consider the communication protocols between nodes, because the design of communication protocols for large-scale WSNs is relatively challenging. In future work, we will further refine the FR-HEX algorithm in deploying nodes, avoiding obstacles and reconstructing the network with more focus on the actual communication situation.

## Figures and Tables

**Table 1:** PCD values of different algorithms at 3000th step in the scale of N=1000 and N=2000.

PCD	*N* = 1000	*N* = 2000
FR-HEX	0.045	0.023
VFA_LJ	0.476	0.584
VFA_DP	0.388	0.484

**Table 2:** Average and standard deviation of execution time of FR-HEX algorithm for 50 simulations with 6000 iterations at different node scales.

Number of Nodes	Average Execution Time (s)	Standard Deviation (s)
N=100	0.286	0.015
N=500	7.025	0.094
N=1000	28.026	0.370
N=2000	113.357	2.081
N=3000	395.961	7.248

**Table 3:** Ratio of the average distance and k in the final deployment state for different conditions.

$\frac{d_{a}}{k}$	*N* = 100	500	1000	1500	2000
a=10	1.426	1.631	1.701	1.735	1.754
a=100	1.426	1.631	1.702	1.735	1.754
